# Comparison of middle molecule removal with expanded hemodialysis versus haemodiafiltration among Chinese hemodialysis patients

**DOI:** 10.1080/0886022X.2025.2498093

**Published:** 2025-05-29

**Authors:** Liangying Gan, Leyi Gu, Yongchun Ge, Hongli Lin, Yiwen Li, Fengling Chen, Wenge Li, Bihu Gao, Feng Ding, Xinzhou Zhang, Zhenwei Shi, Jiayao Ji, Qiang Yao, Brad Keller, Surupa Sarkar, Li Zuo

**Affiliations:** a Peking University People’s Hospital, Beijing, China; b Shanghai Jiao Tong University School of Medicine Affiliated Renji Hospital, Shanghai, China; c Nanjing Jinling Hospital, Nanjing University School of Medicine, Jiangsu, China; d The First Affiliated Hospital of Dalian Medical University, Liaoning, China; e Zhejiang Provincial People’s Hospital, Zhejiang, China; f The First Affiliated Hospital of Soochow University, Jiangsu, China; g China-Japan Friendship Hospital, Beijing, China; h Affiliated Zhongshan Hospital of Dalian University, Liaoning, China; i Shanghai Jiao Tong University School of Medicine Affiliated Ninth People’s Hospital, Shanghai, China; j Shenzhen People’s Hospital, Guangdong, China; k Beijing Huaxin Hospital First Hospital of Tsinghua University, Beijing, China; l Baxter China Investment Company Ltd, Shanghai, China; m Baxter Healthcare Corporation, Deerfield, IL, USA

**Keywords:** Expanded hemodialysis, free light chains, haemodiafiltration, medium cutoff, Chitinase-3-like protein 1

## Abstract

**Background:**

This study aimed to compare uremic toxin removal with expanded hemodialysis against post-dilution online haemodiafiltration therapy in Chinese patients with chronic kidney failure in a single treatment.

**Methods:**

This randomized, controlled, open-label, parallel, multicenter trial enrolled prevalent patients on hemodialysis. The study endpoints were to establish the non-inferiority of expanded hemodialysis versus haemodiafiltration in removing beta-2-microglobulin (β2M) and lambda-free light chains (λFLC) and to evaluate the reduction ratios of urea, alpha-1-microglobulin (α1M), myoglobin, complement factor D, kappa-free light chains (κFLC) and Chitinase-3-like protein 1 (YKL-40) during a mid-week dialysis session. The 95% confidence intervals of the difference in λFLC and β2M reduction ratios with expanded hemodialysis were compared against pre-defined non-inferiority margins (−3.783 and −7.848, respectively). Non-inferior reduction ratios were tested for superiority using hierarchical testing.

**Results:**

Overall, 274 adult patients were randomized to expanded hemodialysis (*n* = 138) or haemodiafiltration (*n* = 136). No differences in demographics, baseline characteristics, and treatment parameters were observed between the arms. The reduction ratio of λFLC with expanded hemodialysis was superior to haemodiafiltration; reduction ratio difference of 17.0% [95% confidence interval: 14.8%, 19.2%]. The reduction ratio of β2M with expanded hemodialysis was non-inferior to haemodiafiltration; reduction ratio difference of −1.2% [95% confidence interval: −2.5%, 0.2%]. Expanded hemodialysis showed significantly higher removal of α1M, YKL-40, complement factor D, myoglobin, and κFLC than haemodiafiltration therapy. There were no significant differences in Kt/V_urea_, urea reduction ratio, and the rate of complications between the arms.

**Conclusion:**

Our study demonstrates the effectiveness of expanded hemodialysis therapy in removing multiple middle molecules compared to haemodiafiltration therapy, with no observed differences in the overall safety of Chinese patients.

## Introduction

It is reported that there are close to 0.9 million maintenance hemodialysis (HD) patients and an annual increment of 9% in China according to the Chinese National Research Data System, which results in a huge disease burden on the public health system [1]. Clinical outcomes in this population remain suboptimal, with recent studies showing that middle molecular toxins with molecular weights ranging from 0.5 to 60 kDa are closely related to inflammation and poor clinical outcomes [2–4]. Therefore, clearing these toxins is necessary and essential to improving patient outcomes and reducing the disease burden.

High-flux HD is a widely used and accessible renal replacement therapy which allows for the clearance of small middle molecules, such as beta-2-microglobulin (β2M). However, high-flux HD does not effectively remove molecules larger than 15 kDa which are linked to vascular calcification, chronic inflammation, immunodeficiency, and an increased risk of cardiovascular morbidity and mortality for patients on dialysis [4–8].

Haemodiafiltration (HDF) is an alternative option that combines diffusion and convection to effectively remove a broader range of middle molecular uremic toxins and has been associated with various clinical benefits [9–11]. However, HDF requires an infrastructure and highly specialized equipment to deliver large volumes of ultrapure dialysate and sterile online replacement fluid. Moreover, HDF is only suitable for patients having well-functioning vascular access allowing for a high blood flow rate [12,13]. In China, it is standard practice to perform HDF therapy once every week or once every two weeks, which is less frequent than in other regions of the world where HDF is used for every session [14]. Additionally, blood flow rates range between 210 and 250 mL/min, and are often applied with a less-than-optimal convective dose [14,15].

The limitation in current dialytic or blood depuration modalities in removing middle molecules required an innovative approach to improving outcomes. A new class of membranes with a cutoff close to the molecular weight of albumin was developed, allowing for high clearances of solutes across a wide spectrum of molecular weights up to 45 kDa [12,16,17]. It was reported that expanded hemodialysis (HDx) enabled by a medium cutoff (MCO) dialyzer has similar or even superior effects to post-dilution online HDF and high-flux HD in clearing middle molecular toxins, however, previous studies comparing these therapies have been limited to small sample sizes primarily representing European populations and clinical practises [16,17]. Yet, treatment parameters for Asian patients, such as lower blood flow and smaller replacement volumes, differ from those in published studies [18]. Thus, it is imperative to determine the effects of HDx enabled by MCO membranes in this specific patient population.

The primary aim of the study was to compare HDx to post-dilution online HDF in terms of the reduction ratio (RR) for various middle-molecular uremic toxins among Chinese HD patients. This study is the largest randomized controlled trial comparing HDx to post-dilution online HDF to date and is the first to evaluate HDx therapy in a Chinese clinical practice setting.

## Materials and methods

This was a randomized, controlled, open-label, parallel and multicenter study. Patients with kidney failure on a stable HD and/or HDF prescription were enrolled and randomized in a 1:1 ratio to receive HDx treatment with Theranova 400 (Baxter Healthcare, Deerfield, IL, USA) or post-dilution online HDF with FX800 (Fresenius Medical Care AG & Co. KGaA, Bad Homburg, Germany) during a single mid-week dialysis session.

The main objective of the study was to investigate the non-inferiority of HDx therapy to post-dilution online HDF therapy in the removal of lambda-free light chains (λFLC) and β2M among Chinese HD patients. Furthermore, the removal of urea, alpha-1-microglobulin (α1M), Chitinase-3-like protein 1 (YKL-40 or human cartilage glycoprotein-39), complement factor D (CFD), myoglobin, and kappa-free light chain (κFLC) were determined. The RR for each uremic toxin was calculated to estimate its removal using the following formula: [(C_pre_–C_post_)/C_pre_], where C_pre_ and C_post_ were the arterial plasma concentrations measured pre and post the mid-week dialysis session, respectively [19]. For λFLC, κFLC, α1M, CFD, YKL-40, myoglobin and β2M, C_post_ was corrected for the decrease in total extracellular volume due to fluid removal [20]. Single pool Kt/V_urea_, and urea reduction ratio (URR) were assessed to establish dialysis adequacy [21]. Safety assessments included treatment arm comparability of the incidence of adverse events, serious adverse events and device deficiencies.

The key criteria for inclusion in this study were that patients had to be ≥ 18 years old and ≤ 80 years old, were able to provide consent for participation, were stable receiving in-centre HD or HDF for more than 3 months before study enrollment, had an adequate arteriovenous fistula or graft, or dual-lumen tunneled catheter capable of providing a blood flow rate (QB) of at least 250 mL/min, had a minimum total convective volume (including ultrafiltration [UF]) of 16 L post-dilution during the most recent HDF treatment before enrollment, and had Kt/V_urea_ > 1.2 for the last 2 measurements with the most recent Kt/V_urea_ measurement taken within 4 weeks before or during study screening. There were no gender restrictions for this study. Patients were excluded if there were safety concerns around their ability to participate.

A full list of all inclusion and exclusion criteria is provided alongside the study’s trial database registration (ClinicalTrials.gov ID: NCT05309291; registered on 25 March 2022).

### Ethical considerations

This study was conducted according to the requirements of the National Medical Products Administration (NMPA) and National Health and Family Planning Commission (NHFPC), Good Clinical Practice (GCP) for Medical Devices, the GCP guidelines of the International Council for Harmonization (ICH) and followed the ethical principles specified in the Declaration of Helsinki. The study was performed after receiving approval by the ethics committees and written informed consent was obtained from all patients prior to participation.

### Statistical analyses

The statistical hypothesis for testing the treatment group difference for RR of λFLC or β2M is presented as follows:

$$\text{Null hypothesis }{\text{(H}}_{0}\text{): }\mu T-\mu R\leq -\delta$$

$$\text{Alternative hypothesis }{\text{(H}}_{a}\text{): }\mu T-\mu \text{R > }-\delta$$

whereby μT represents the HDx treatment mean, μR is the HDF treatment mean, and δ is the non-inferiority margin.

A t-test was used to generate a two-sided 95% confidence interval (CI) for the difference in means (μT – μR). The non-inferiority margin for λFLC was −3.783 (i.e., 10% of an assumed mean RR of 37.83%) and −7.848 (i.e., 10% of an assumed mean RR of 78.48%) for β2M. If the lower bound of each CI exceeds the margin, then non-inferiority can be established. If the lower bound of each CI > 0, then the superiority of HDx treatment over HDF treatment can be concluded. A hierarchical structure was used for testing both non-inferiority and superiority. Superiority was only tested if non-inferiority passed. A t-test or Wilcoxon Rank-Sum test, as applicable, was used to evaluate differences between treatment arms in Kt/V_urea_, URR, and the RRs of α1M, YKL-40, CFD, myoglobin and κFLC. Treatment arm comparability of the incidence of adverse events was evaluated using Fisher’s exact test. Data are presented as mean (±standard deviation [SD]) unless indicated otherwise.

## Results

### Patient population

**Figure 1.**
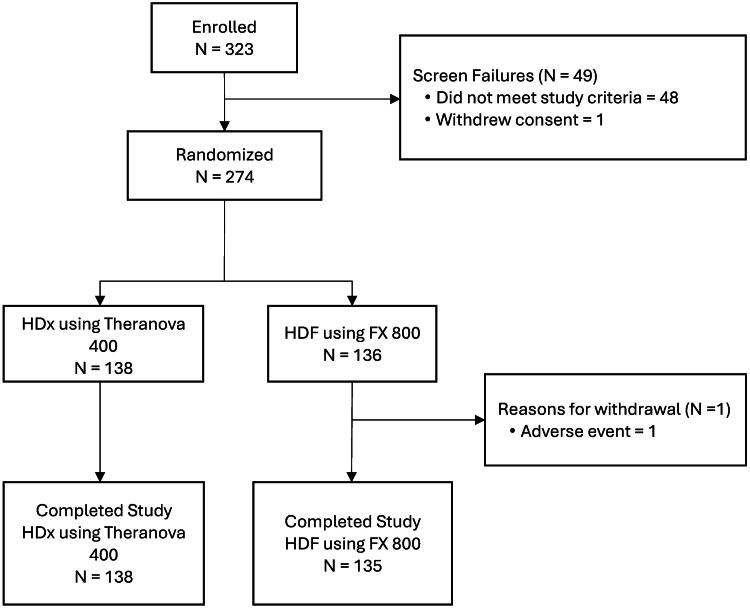
Patient disposition.

A total of 323 patients across 11 centers in China were eligible for enrollment between June 2022 and July 2023. Forty-nine (15.2%) patients failed screening (14.9% did not meet the study criteria and 0.3% withdrew consent) while the remaining 274 patients were randomized, either to HDx therapy (*n* = 138) or to HDF therapy (*n* = 136). Before completing the study, 1 patient was withdrawn from the HDF arm due to an adverse event (device allergy); therefore, 138 patients in the HDx arm and 135 patients in the HDF arm completed the study (Figure 1).

The age of the study population was 52.6 (±11.7) years, ranging from 22 to 77 years. There was a higher representation of males in the total population (71.2%) as well as in each of the cohorts (68.1% in the HDx arm and 74.3% in the HDF arm). The dialysis vintage of all patients was 6.7 (±4.7) years (Table 1). None of the treatments were interrupted during the study. The average utilized replacement volume in the HDF arm was 16.7 (±2.4) L and the ultrafiltration rate was 10.9 (±7.6) mL/min. Treatment parameters measured during the mid-week dialysis session were comparable between both arms (Table 2).

**Table 1.** Demographics and baseline characteristics.

Characteristic	Value	HDx *n* = 138	HDF *n* = 136	Total *n* = 274
Age, years	Mean (SD)	52.1 (12.0)	53.0 (11.4)	52.6 (11.7)
Dry weight, kg	Mean (SD)	62.5 (10.4)	64.0 (11.0)	63.2 (10.7)
BMI, kg/m^2^	Mean (SD)	23.0 (3.2)	23.2 (3.2)	23.1 (3.2)
Sex, *n* (%)	Male	94 (68.1)	101 (74.3)	195 (71.2)
	Female	44 (31.9)	35 (25.7)	79 (28.8)
Ethnicity, *n* (%)	Chinese	138 (100)	135 (99.3)	273 (99.6)
	Unknown	0	1 (0.7)	1 (0.4)
Dialysis vintage, years	Mean (SD)	7.17 (4.86)	6.28 (4.45)	6.73 (4.67)

Baseline is defined as the last non-missing measurement before the first application of the study device.

Analysis based on the number of patients, as randomized.

BMI: body mass index; HDF: hemodiafiltration; HDx: expanded hemodialysis; SD: standard deviation.

**Table 2.** Treatment parameters at the mid-week dialysis session.

Characteristic	Value	HDx *n* = 139	HDF *n* = 135	Total, *n* = 274
Total dialysis time, min	Mean (SD)	240.0 (1.5)	241.0 (9.5)	240.5 (6.8)
QB, mL/ min	Mean (SD)	261.6 (10.2)	269.4 (17.7)	265.4 (14.9)
QD, mL/min	Mean (SD)	506.3 (30.70)	526.0 (68.09)	516.1 (53.45)
QUF, mL/min	Mean (SD)	10.5 (6.73.19)	10.9 (7.64)	10.7 (5.82)
Pre-dialysis body weight, kg	Mean (SD)	65.0 (10.58)	66.5 (11.26)	N/A
Post-dialysis body weight, kg	Mean (SD)	62.6 (10.28)	64.1 (11.02)	N/A
Type of vascular access, *n* (%)	Permanent tunneled-cuffed dialysis catheter	1 (0.7)	1 (0.7)	2 (0.7)
	Arteriovenous fistula	137 (98.6)	134 (99.3)	271 (98.9)
	Arteriovenous graft	1 (0.7)	0	1 (0.4)
Post-dilution replacement volume, L	Mean (SD)	N/A	16.7 (2.40)	16.7 (2.40)
Treatment interruption, *n* (%)	No	139 (100)	135 (100)	274 (100)
	Yes	0	0	0

Analyses based on the number of patients, as treated.

HDF: haemodiafiltration; HDx: expanded hemodialysis; QB: blood flow rate; QD: dialysis fluid flow rate; QUF: ultrafiltration flow rate; SD: standard deviation.

### Efficacy outcomes

The RR of λFLC in the HDx arm was 36.0 (±9.6) % [95% CI: 34.4%, 37.7%] and 19.0 (±8.0) % [95% CI: 17.6%, 20.4%] in the HDF arm, giving a difference in means of 17.0 (±8.9) % [95% CI: 14.8%, 19.2%]. The RR of λFLC in the HDx arm was superior to the HDF arm (*p* < .0001) since the lower bound of the CI (14.8%) exceeded the non-inferiority margin of −3.783 and was greater than zero. The RR of β2M in the HDx arm was 77.0 (±5.1) % [95% CI: 76.2%, 77.9%] and 78.2 (±6.0) % [95% CI: 77.2%, 79.3%] in the HDF arm, giving a difference in means (SD) of −1.2 (±5.6) % [95% CI: −2.5%, 0.2%]. The RR of β2M in the HDx arm was demonstrated to be non-inferior to the HDF arm (*p* < .0001) since the lower bound of the 95% CI (−2.5%) was higher than the non-inferiority margin of −7.848 (Table 3).

**Table 3.** Summary and analyses of RRs of λFLC and β2M.

	HDx *n* = 131	HDF *n* = 131	Difference HDx vs. HDF	*p* value
*Reduction Ratios of λFLC, %*				
Mean (SD)	36.0 (9.62)	19.0 (8.02)	17.0 (8.9)	–
95% CI	34.4, 37.7	17.6, 20.4	14.8, 19.2	<.0001
*Reduction Ratios of β2M, %*				
Mean (SD)	77.0 (5.10)	78.2 (5.97)	−1.2 (5.55)	–
95% CI	76.15, 77.91	77.19, 79.25	−2.54, 0.16	<.0001

Analyses are based on the number of patients randomized and who did not have any major protocol deviations that could affect the assessment of the primary endpoint (Major protocol deviations included: actual QB < 220 mL/min for more than 30 or 60 min at the mid-week treatment day dialysis session; patients in the control group who did not achieve at least 16 L convective volume (including UF); actual dialysis duration <210 min at the mid-week treatment day dialysis session).

β2M: beta 2 microglobulin; CI: confidence interval; HDF: haemodiafiltration; HDx: expanded hemodialysis; λFLC: lambda free light chain; SD: standard deviation.

**Figure 2.**
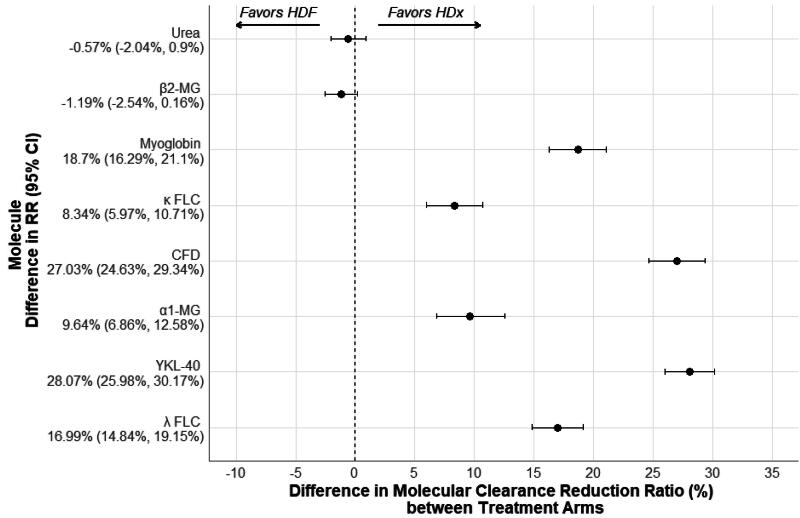
The difference in molecular reduction ratio (%) between treatment arms.

There were no significant differences in dialysis adequacy Kt/V_urea_ (*p* = .3674) and URR (*p* = .4475) between the treatment arms. The HDx arm had a higher mean RR for myoglobin and for κFLC (*p* < .0001, each) and a higher median RRs for α1M, YKL-40, and CFD compared to the HDF arm (*p* < .0001, each) (Table 4 and Figure 2).

**Table 4.** Summary and analyses of dialysis adequacy and the RRs of other Middle molecules.

	HDx *n* = 138	HDF *n* = 136	Difference HDx vs. HDF	*p* value
*Dialysis Adequacy, Kt/V_urea_*				
Mean (SD)	1.55 (0.26)	1.58 (0.27)	−0.03 (0.27)	–
95% CI	1.51, 1.60	1.54, 1.63	−0.09, 0.03	.3674
*Reduction Ratios of Urea*, %				
Mean (SD)	72.18 (5.73)	72.75 (6.59)	−0.57 (6.18)	–
95% CI	71.21, 73.15	71.62, 73.87	−2.04, 0.90	.4475
*Reduction Ratios of α1M*, %				
Median	23.21	13.15	9.64	–
95% CI	–	–	6.86, 12.58	<.0001
*Reduction Ratios of YKL-40*, %				
Median	57.60	29.24	28.07	–
95% CI	–	–	25.98, 30.17	<.0001
*Reduction Ratios of CFD*, %				
Mean (SD)	56.71 (16.20)	30.24 (19.15)	–	–
95% CI	–	–	24.63, 29.34	<.0001
*Reduction Ratios of Myoglobin*, %				
Mean (SD)	64.67 (7.24)	45.97 (12.37)	18.70 (10.10)	–
95% CI	63.45, 65.89	43.87, 48.08	16.29, 21.10	<.0001
*Reduction Ratios of κFLC*, %				
Mean (SD)	58.19 (8.53)	49.85 (11.02)	8.34 (9.84)	–
95% CI	56.73, 59.64	47.96, 51.73	5.97, 10.71	<.0001

Analyses based on the number of patients, as randomized.

α1M: alpha-1 microglobulin; CI: confidence interval; CFD: complement factor D; HDF: haemodiafiltration; HDx: expanded hemodialysis; κFLC: kappa free light chain; SD: standard deviation, YKL-40: Chitinase-3-Like Protein 1/Human Chitinase Glycoprotein.

### Safety outcomes

Eighteen patients experienced a total of 19 adverse events. These included events of thrombosis, dizziness, hypotension, hypertension, device allergy, arteriovenous fistula occlusion, thrombocytopenia, and hypokalemia. One adverse event (device allergy) in the HDF arm led to the patient’s withdrawal from the study. Thirteen events were mild and six were moderate in severity. The overall incidence of adverse events was 6.6%. None of the treatment-emergent complications were severe and there was no significant difference in the incidence of treatment-emergent adverse events between the treatment arms (4.3% in the HDx arm versus 8.9% in the HDF arm) (*p* = .1481) (Table 5).

**Table 5.** Safety outcomes.

	HDx *n* = 139		HDF *n* = 135		Overall, *n* = 274		
	*n* (%)	Events	*n* (%)	Events	*n* (%)	Events	*p* value ^*^
Any TEAE	6 (4.3)	7	12 (8.9)	12	18 (6.6)	19	.1481
Mild^b^	4 (2.9)		8 (5.9)	8	12 (4.4)	13	–
Moderate^c^	2 (1.4)	2	4 (3.0)	4	6 (2.2)	6	–
Severe^d^	0	0	0	0	0	0	–
Treatment-Related TEAE	2 (1.4)	2	4 (3.0)	4	6 (2.2)	6	.4421
Procedure-Related TEAE	3 (2.2)	4	5 (3.7)	5	8 (2.9)	9	.4959
Serious AEs	1 (0.7)	1	0	0	1 (0.4)	1	1.0000
AEs leading to withdrawal from the study	0	0	1 (0.7)	1	1 (0.4)	1	.4927
Treatment-Related AEs leading to withdrawal	0	0	1 (0.7)	1	1 (0.4)	1	.4927

Analyses based on the number of patients, as treated.

AE: adverse event; HDF: haemodiafiltration; HDx: expanded hemodialysis; TEAE: treatment-emergent adverse event.

*p value was provided by Fisher exact test.

^a^Transient, mild, no influence on daily life and activities, no special measures or treatment required.

^b^Slight influence on daily life and activities, and measures or treatment should be taken when necessary.

^c^Seriously affecting daily life and activities, special measures or treatment must be taken, and hospitalization is required when necessary, which may be life-threatening.

One serious adverse event (arteriovenous fistula occlusion) was reported in HDx arm. The patient underwent an autologous arteriovenous fistula thrombectomy and reconstruction and had fully recovered from the procedure by the next follow-up visit. None of the patients died during the study and no device deficiencies were reported.

## Discussion

This study is the first to compare HDx with HDF in Chinese HD patients and shows that HDx is non-inferior or superior to HDF in clearing middle molecules in the our clinical setting.

This study provides a unique representation of the practice, adaption, and implementation of HDF in China, which differs from European practices. During this study, the mean blood flow rate of patients in the HDF group was 269.4 mL/min, which is higher than the value reported in the Chinese DOPPS 5 study (230 mL/min), but meets the requirements of the Standard Operating Procedure for Blood Purification (2021 version) (>250 mL/min) [15]. The mean replacement fluid volume in the post-dilution HDF group was 16.7 L and the mean difference in pre- and post-dialysis body weight for this group was 2.36 kg, which is related to the net sum of fluid removed during dialysis treatment (i.e., ultrafiltration volume) at a measured rate of 10.9 (±7.6) mL/min. Combined, the total convection volume, which is the sum of the substitution fluid volume and the net ultrafiltration volume, achieved in this study for a single treatment in the post-dilution HDF group was approximately 19 L. This is comparable to the 17.2 ± 1.3 L convective volume recorded for the online HDF patients in the Turkish OL-HDF Study [22–24] but is significantly higher than the post-dilution HDF convective volume published in Japan and lower than the 22 to 26 L convection volumes reported elsewhere [25–27].

HDx is better than HDF in clearing large middle molecular toxins in Chinese clinical practice. Lambda-free light chains (λFLC) (45 kDa) were one of the molecules selected to evaluate the primary objective of the study. As a large middle molecular toxin, it is an important biomarker of immune dysfunction and inflammation [28]. Studies have shown that λFLC is markedly elevated in dialysis patients and is associated with inflammation, vascular calcification, disease progression, and increased risk of death [5,6,29]. Our results showed a superior RR for λFLC with HDx than HDF, which is comparable with the outcomes reported in the studies by Kirsch et al. and Kim et al. [16,18]. Two additional large middle molecular toxins, YKL-40 and α1M, were assessed as secondary endpoints. YKL-40 has a molecular weight of 40 kDa. It is secreted by activated macrophages and other cells and is induced by pro-inflammatory cytokines. It is known to regulate other inflammatory markers and is associated with the risk of cardiovascular events and death in dialysis patients [30]. α1M has a molecular weight of 33 kDa [7,31]. Sakurai reported that a reduction in α1M levels was associated with reduced symptoms of restless legs syndrome in HD patients [32]. Similar to previous studies, the HDx treatment arm in our study showed significantly higher median RRs of YKL-40 and α1M when compared to the HDF treatment arm [16,33–36].

HDx is equivalent or superior to HDF in removing small, and medium middle molecular toxins in our clinical practice. Another molecule evaluated for the primary endpoint of the study was β2M, a polypeptide with a molecular weight of 11.8 kDa which has been extensively used as a marker for small middle molecule removal and which is associated with cardiovascular disease [12,37]. Studies comparing the RR of β2M with HDx to post-dilution HDF show mixed results [38,39]. In a meta-analysis published by Zhao et al., 9 studies compared HDx with HDF [39]. These showed that the RR of β2M in the HDx group was significantly higher than that in the HD group, but lower than that in the HDF group. On the other hand, the study by Kirsch et al. showed that patients in the HDx group had a higher clearance than the HDF group [40]. Maduell et al. showed that the RR for β2M was comparable between HDx and 8 different high-flux dialyzers in high-volume post-dilution online HDF [36]. These findings are probably related to the higher convective volume delivered in addition to some contribution to diffusive transfer. Additionally, the HDx treatment arm in our study showed a significantly higher RR of myoglobin, κFLC and CFD when compared to the HDF treatment arm, which concurs with the outcome of other published studies [18,34,40,41]. It was reported that HDx is able to deliver internal convection of 30–53 mL/min, depending on blood flow rate and model size, which approximately amounts to 7.2–12.7 L convective volume during a 4-h session [42]. The combination of the enhanced sieving properties of the medium cutoff membrane and the avoidance of high filtration fractions enables equal or superior convective removal of middle molecules during HDx compared to HDF [43].

### Limitations

This trial focused on the mid-week dialysis session for patients in each treatment arm, which precludes its ability to evaluate clinical outcomes, such as morbidity and mortality as well as the changes in pre-dialysis uremic toxin levels or serum albumin level. Although published studies have shown that thrice-weekly HDx will not reduce the patient’s serum albumin level [17,44], an additional long-term study is probably needed to verify this in Chinese HD patients. In addition, the study did not detect the loss of albumin in dialysate due to complicated methodology, which is not feasible in a large sample size study as such.

Due to the limitation of medical resources and economic factors, Chinese patients usually undergo HDF once a week. Therefore, the dialysis mode before mid-week HDF in the study was mostly HD, which makes the study different from most studies that have compared HDx to HDF. Among all the uremic toxins, protein-bound toxins are not studied which need future studies to answer related questions.

Despite the above limitations, this is the first randomized study of HDx among Chinese patients in comparison to HDF. This provides the first comparative study of middle molecule removal in this population. It also highlights the need to perform additional long-term trials to demonstrate the effects of this emerging therapy in China.

## Conclusion

Our study demonstrates the effectiveness of HDx therapy in clearing multiple middle molecules when compared to HDF therapy, with no observed differences in the overall safety of Chinese patients. The outcome of this study suggests that HDx can be used as a safe and effective alternative for patients who are unable to perform HDF. Nevertheless, long-term follow-up studies are essential to confirm this observation.

## Data Availability

Anonymized individual patient datasets and supporting documents (synopsis of the clinical study report, protocol, and SAP) will be made available upon approval of a legitimate research request. Research requests will be reviewed by qualified medical and scientific experts. If an agreement to the release of clinical data for research purposes is made, the requestor will be required to sign a data-sharing agreement to ensure the protection of patient confidentiality and any intellectual property rights. Data will be made available beginning 9 months and ending 36 months after publication. Proposals should be submitted to the corresponding author. This study has been registered on ClinicalTrials.gov, a publicly accessible database (NCT05309291).
